# Increase in invasive group A streptococcal (*Streptococcus pyogenes*) infections (iGAS) in young children in the Netherlands, 2022

**DOI:** 10.2807/1560-7917.ES.2023.28.1.2200941

**Published:** 2023-01-05

**Authors:** Brechje de Gier, Niek Marchal, Ilse de Beer-Schuurman, Margreet te Wierik, Mariëtte Hooiveld, ISIS-AR Study Group, GAS Study group, Hester E. de Melker, Nina M. van Sorge, Members of the ISIS-AR study group, J.W.T. Cohen Stuart, D.C. Melles, K. van Dijk, A. Alzubaidy, M. Scholing, S.D. Kuil, G.J. Blaauw, W. Altorf-van der Kuil, S.M. Bierman, S.C. de Greeff, S.R. Groenendijk, R. Hertroys, E.J. Kuijper, N. Marchal, J.C.M Monen, D.W. Notermans, J. Polman, W.J. van den Reek, C. Schneeberger–van der Linden, A.F. Schoffelen, C.C.H. Wielders, B.J. de Wit, R.E. Zoetigheid, W. van den Bijllaardt, E.M. Kraan, M.B. Haeseker, J.M. da Silva, E. de Jong, B. Maraha, A.J. van Griethuysen, B.B. Wintermans, M.J.C.A. van Trijp, M. Wong, A.E. Muller, A. Ott, M. Lokate, E. Bathoorn, J. Sinnige, D.C. Melles, W. Silvis, N.H. Renders, L.J. Bakker, J.W. Dorigo-Zetsma, K. Waar, M.T. van der Beek, M.A. Leversteijn-van Hall, S.P. van Mens, E. Schaftenaar, M.H. Nabuurs-Franssen, I. Maat, B.M.W. Diederen, L.G.M. Bode, D.S.Y. Ong, M. van Rijn, S. Dinant, O. Pontesilli, D.W. van Dam, E.I.G.B. de Brauwer, R.G. Bentvelsen, A.G.M. Buiting, A.L.M. Vlek, M. de Graaf, A. Troelstra, A.R. Jansz, M.P.A. van Meer, J. de Vries, C. Oliveira dos Santos, Members of the GAS study group, Lidewij W. Rümke, Stefan M. T. Vestjens, Bart J.M. Vlaminckx

**Affiliations:** 1 Center for Infectious Disease Control, National Institute for Public Health and the Environment (RIVM), Bilthoven, the Netherlands; 2 Netherlands Reference Laboratory for Bacterial Meningitis, Amsterdam University Medical Center location AMC, Amsterdam, the Netherlands; 3 Nivel, Utrecht, the Netherlands; 4 Members of the ISIS-AR Study Group are listed under Collaborators.; 5 Members of the GAS Study Group are listed under Collaborators.; 6 Department of Medical Microbiology and Infection Prevention, Amsterdam UMC location University of Amsterdam, Amsterdam Institute for Infection and Immunity, Amsterdam, the Netherlands

**Keywords:** *Streptococcus pyogenes*, iGAS, varicella

## Abstract

In 2022, a sevenfold increase in the number of notifiable invasive *Streptococcus pyogenes* (iGAS) infections among children aged 0–5 years was observed in the Netherlands compared with pre-COVID-19 pandemic years. Of 42 cases in this age group, seven had preceding or coinciding varicella zoster infections, nine were fatal. This increase is not attributable to a specific *emm* type. Vigilance for clinical deterioration as iGAS sign is warranted in young children, especially those with varicella zoster infection.

A more than twofold increase in the annual number of notifiable invasive group A streptococcal (iGAS) infections compared to the annual average in the pre-COVID-19 pandemic years was observed in the Netherlands in 2022. This increase was even higher children aged 0-5 years, with 42 iGAS notifications in this age group in 2022. We here describe the epidemiological situation of iGAS infections in 2022 using several complementary data sources.

## National invasive group A streptococcal infection notification

In the Netherlands, three clinical presentations of culture-confirmed iGAS are notifiable by law. Streptococcal toxic shock syndrome (STSS) and necrotising fasciitis are notifiable to enable public health officials to provide antibiotic prophylaxis to close contacts. Puerperal fever or -sepsis is notifiable to enable source tracing in case of transmission by healthcare workers.

**Figure 1:**
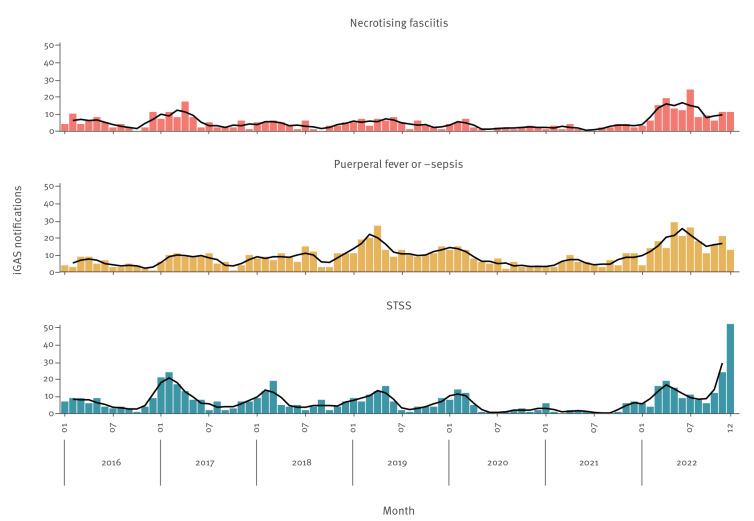
Notifications of culture-confirmed invasive group A streptococcal infection (iGAS) by disease presentation and month of disease onset, for all ages, the Netherlands, 1 January 2016–31 December 2022 (n = 1,816)

Notifications of non-puerperal iGAS (STSS or necrotising fasciitis) were markedly increased in 2022 (n = 319) compared with four pre-COVID-19 pandemic years 2016–19 (annual mean: 146) and after two pandemic years with relatively few notifications (81 and 51, respectively) (Figure 1). While the number of necrotising fasciitis cases was most notably increased in the first half of 2022, STSS cases increased steeply at the end of the year. Of the 319 non-puerperal iGAS cases notified in 2022, the median age was 55 (interquartile range (IQR): 34-68) years, 149/319 (47%) were female and 170/319 (53%) were male. Cases were geographically spread across the country.

The black lines represent three-month moving averages.

**Figure 2:**
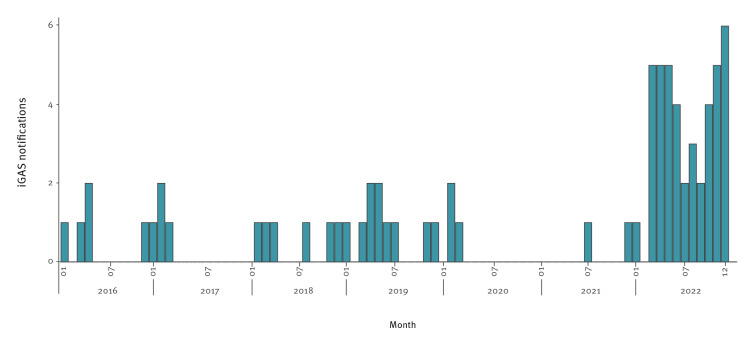
Notifications of culture-confirmed invasive group A streptococcal infections (streptococcal toxic shock syndrome or necrotising fasciitis) among children aged 0–5 years, by month of disease onset, the Netherlands, 1 January 2016–31 December 2022 (n = 72)

For children aged 0–5 years, the increase compared with pre-COVID-19 pandemic years was even more striking (Figure 2). Between 2016 and 2019, the proportion of non-puerperal iGAS notifications that occurred among children aged 0–5 years ranged between 2% and 7%. In 2022, this proportion was 13%. In absolute numbers, the annual iGAS notifications in children aged 0–5 years increased sevenfold with 42 notifications compared with on average six cases in 2016–19, and three and two in 2020 and 2021, respectively. Among the 42 iGAS notifications in this age group in 2022, seven reported a varicella zoster infection preceding or coinciding with iGAS infection. As a varicella zoster infection is not part of the standard questionnaire for notification, this is likely an underestimation of the true number of coinfections. Moreover, nine of the 42 notified iGAS infections in young children were reported as fatal. This might also be an underestimation, as it is not mandatory to follow-up an iGAS notification with clinical outcomes.

## Bacteriological surveillance and *emm* typing

The Dutch National Reference Laboratory for Bacterial Meningitis (NRLBM) at the Amsterdam University Medical Center, offers *emm* typing for *S. pyogenes* isolates submitted by microbiological laboratories. Since 2019, bacteriological surveillance of iGAS infections was initiated as part of a pilot study. Nine laboratories (covering ca 28% of the Dutch population) have voluntarily submitted all *S. pyogenes* isolates cultured from normally sterile compartments for *emm* typing to the NRLBM. Since May 2022, the NRLBM and the Dutch National Institute for Public Health and the Environment (RIVM) have requested all microbiological laboratories to submit *S. pyogenes* isolates cultured from normally sterile sites for *emm* typing. Submission is on a voluntary basis.

**Figure 3:**
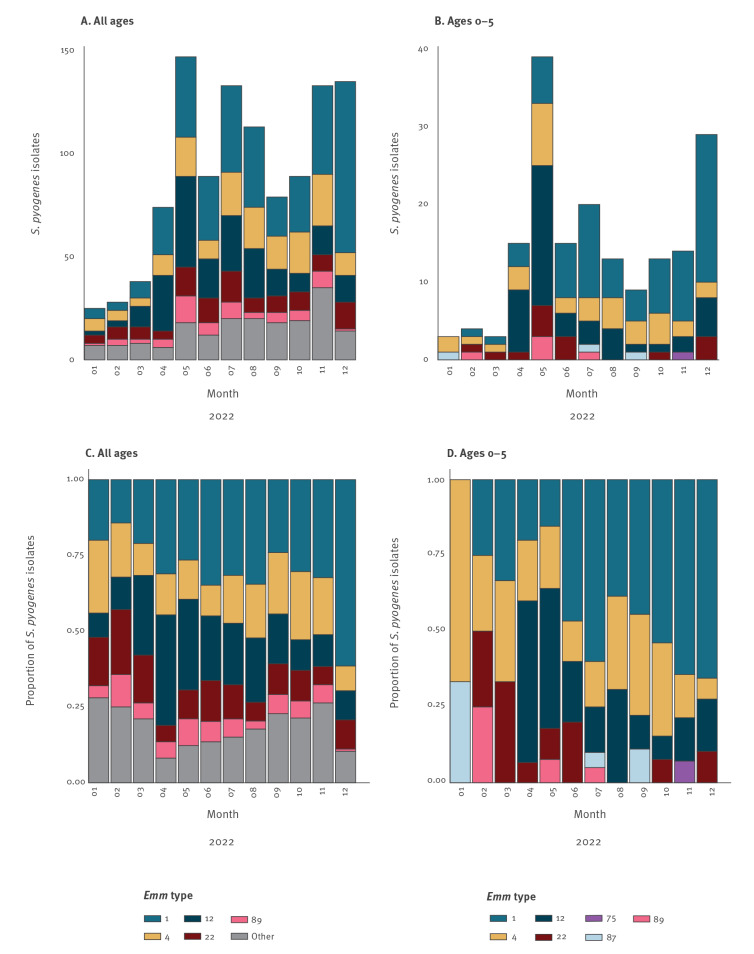
(A) Number and (C) proportion of *emm* types among all *Streptococcus pyogene*s isolates (n = 1,083) and (B) number and (D) proportion among *S. pyogene*s isolates from children aged 0–5 years (n = 177), by month of isolate submission to NRLBM, the Netherlands, 1 January–31 December 2022

Figure 3 shows the *emm* types of *S. pyogenes* isolates typed in 2022, by month, for all ages (Figure 3A) and children aged 0–5 years (Figure 3B). The total numbers of 1,083 (all ages) and 177 (ages 0–5) are much higher than in the 2022 notifications in Figure 1 and 2, because typing results also include non-notifiable manifestations of iGAS infection, such as meningitis or sepsis. From May 2022, the number of typed isolates has strongly increased since isolates are received from all medical microbiology laboratories following a national request by NRLBM and RIVM on 10 May. The data show that there is not one specific *emm* type responsible for the high incidence among young children. Nonetheless, *emm* types 1, 4, 12, 22 and 89 make up >80% of the pathogenic isolates, both for all ages (899/1,083) and for children aged 0–5 years (173/177). The proportion of *emm*1 among isolates from children aged 0–5 years was larger between June and December 2022, compared with January to May 2022. Compared with typed isolates from 2019 (data not shown), the contribution of *emm*4 is most notable, increasing from 6% to 20% in children aged 0–5 years.

NRLBM: Dutch National Reference Laboratory for Bacterial Meningitis.

From May 2022, the number of typed isolates has strongly increased since isolates were received from all medical microbiology laboratories following a national request by the NRLBM and RIVM. Underlying data, including separate *emm* types here aggregated as ‘other’, are available in Table S1.

## Additional data sources

### Dutch medical microbiology laboratories

**Figure 4:**
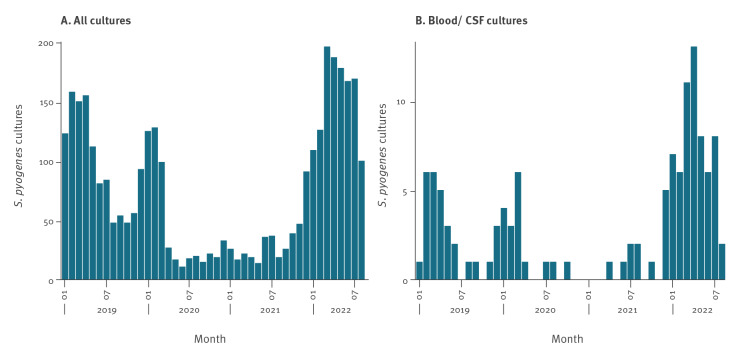
Number of children aged 0–5 years with a positive *Streptococcus pyogene*s culture per month, (A) all diagnostic (infection-related) cultures (n = 3,321) and (B) cultures from blood or cerebrospinal fluid (n =119), from 36 medical microbiological laboratories, the Netherlands, January 2019–August 2022

All Dutch medical microbiology laboratories routinely perform antimicrobial susceptibility testing on iGAS isolates. The national surveillance system of antimicrobial resistance (ISIS-AR) collects data of all cultures for which an antibiotic susceptibility test was performed for the larger part of the country [1]. Using data from 36 medical microbiological laboratories supplying complete data from January 2019 to August 2022, the number of children aged 0–5 years from whom at least one *S. pyogenes* isolate was cultured was analysed. For children with more than one *S. pyogenes* isolate, only the first isolate was included. The number of children with a *S. pyogenes* positive culture between January and August was higher in 2022 compared to 2019, for all diagnostic (infection-related) cultures (n = 1,232 in 2022, n = 911 in 2019)(Figure 4A), with a stronger increase for cultures specifically from blood or cerebrospinal spinal fluid, indicating invasive infection (n = 61 in 2022, n = 24 in 2019) (Figure 4B). The number of *S. pyogenes* isolates exceeded the number of notifications in Figure 2. This indicates that the increased disease burden from iGAS among young children is not restricted to the notifiable disease presentations STSS and necrotising fasciitis but extends to other disease manifestations.

CSF: cerebrospinal spinal fluid.

Please note the different scaling for panels A and B.

Previous ISIS-AR analyses have shown increasing resistance of *S. pyogenes* to clindamycin and erythromycin in the Netherlands during 2017–21 (from 4% to 9% resistance to clindamycin and from 6% to 11% resistance to erythromycin among hospital isolates, respectively) [2].

### Nivel Primary Care Database

Nivel's Primary Care Database uses routinely recorded data from healthcare providers to monitor health and utilisation of health services in a representative sample of the Dutch population. In this Primary Care Database, a relatively high number of general practitioner (GP) consultations with diagnosis ICPC-code R72 (strep throat or scarlet fever) was noted for children aged 5–14 years during 2022 (approximately twice as high as in 2019) [3,4]. In the first half of 2022, the number of GP consultations for varicella zoster among children 0–14 years old was also notably high, and about twofold higher compared with 2016–19 for children aged 5–14 years (Figure S1). Among the seven children aged 0–5 years notified with varicella zoster preceding or coinciding with iGAS, the median age was 2 years (range: 1–5). This corresponds to the median age of varicella zoster infections in the Netherlands in the pre-COVID-19 era, and therefore does not indicate an age shift in varicella infection as possible explanation for the increased risk of iGAS, in line with a previous German study [5,6].

## Discussion

Early December 2022, the United Kingdom Health Security Agency (UKHSA) published a surveillance report on scarlet fever and iGAS and issued a warning to parents and clinicians about the high iGAS incidence among children [7]. From 12 September to 4 December, 13 children (<18 year-olds) died from iGAS in the United Kingdom (UK).

*Streptococcus pyogenes*, often synonymously referred to as group A *Streptococcus* (GAS), can be carried asymptomatically in the nasopharynx and on skin but can also cause a wide array of diseases. These include both non-invasive diseases such as impetigo, pharyngitis and scarlet fever, as well as life-threatening invasive disease, which can present among others as sepsis, meningitis, necrotising fasciitis or puerperal sepsis. STSS can develop as a severe complication of iGAS infection, with a high case fatality rate (37.9–59% in previous studies) [8,9].

After the communication from the UK, the European Centre for Disease Prevention and Control and the World Health Organization Regional Office for Europe consulted with several countries conducting iGAS surveillance, and noted paediatric iGAS increases in France, Ireland and Sweden as well [10,11]. The three countries reported increases particularly in the second half of 2022, while paediatric iGAS in the Netherlands has increased since early 2022. These observations in several countries could indicate that the increases in paediatric iGAS are attributable to a large pool of susceptible individuals, especially children, that has developed as a consequence of reduced exposure to GAS and/or other predisposing infections such as varicella zoster and other respiratory viruses in times of physical distancing during the COVID-19 pandemic. After 2 years of low incidence, resurgence of predisposing viral infections may have amplified the resurgence of iGAS infections. A survey by Dutch paediatricians found 16 varicella zoster infections and nine influenza infections among 49 paediatric iGAS cases between July 2021 and June 2022, highlighting the role of viral infection in the risk of iGAS [12]. Because preceding viral infections were not part of the standard iGAS notification questionnaire, we cannot reliably estimate the contribution of such viral infections on the iGAS incidence. From January 2023, a question on symptoms compatible with, and laboratory confirmation of, viral infection preceding iGAS has been added to the notification questionnaire that should allow us to assess the extent of notifiable iGAS disease preceded by viral infections in the coming months.

## Conclusions

Regardless of the epidemiological explanations for our observations, the ongoing increase, combined with the severity and case fatality rate of iGAS, warrants awareness among GPs and other clinicians, public health officials and parents. More specifically, clinical deterioration in children with chickenpox or respiratory viral infection should raise suspicion of a possible *S. pyogenes* superinfection warranting early antibiotic treatment and, where indicated, hospital admission.

## Supplementary Data

312000 Supplementary Material
