# Supplementary material for: Increase in invasive group A streptococcal (Streptococcus pyogenes) infections (iGAS) in young children in the Netherlands, 2022
Source: Euro Surveill. 2023 Jan 5;28(1):2200941. doi: 10.2807/1560-7917.ES.2023.28.1.2200941 (PMC9817208; doi:10.2807/1560-7917.ES.2023.28.1.2200941)
Supplement: Supplementary Material [file 22-00941_SupplementaryMaterial.pdf]

Supplementary material

This supplementary material is hosted by Eurosurveillance as supporting information alongside the article *Increase in invasive group A streptococcal (Streptococcus pyogenes) infections (iGAS) in young children in the Netherlands, 2022* on behalf of the authors who remain responsible for the accuracy and appropriateness of the content. The same standards for ethics, copyright, attributions and permissions as for the article apply. Supplements are not edited by Eurosurveillance and the journal is not responsible for the maintenance of any links or email addresses provided therein.

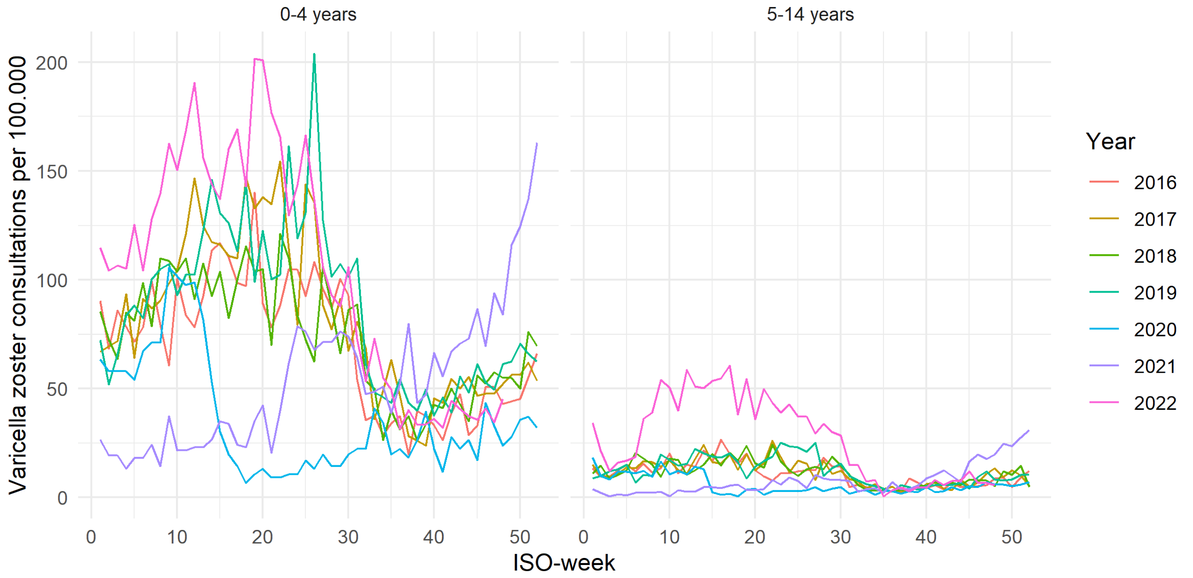

Figure S1. General practitioner consultations per 100.000 inhabitants, ICPC code A72 (varicella zoster infection), Week 1 2016- week 48 2022.

Table S1. Number of S.pyogenes isolates per emm type and month of isolate submission, 2022, all ages.

Month	*emm* type	n
**2022-01**	1	5
**2022-01**	4	6
**2022-01**	8	1
**2022-01**	12	2
**2022-01**	22	4
**2022-01**	28	3
**2022-01**	87	1
**2022-01**	89	1
**2022-01**	102	2
**2022-02**	1	4
**2022-02**	4	5
**2022-02**	12	3
**2022-02**	22	6
**2022-02**	27	1

**2022-02**	28	1
**2022-02**	60	1
**2022-02**	83	1
**2022-02**	87	2
**2022-02**	89	3
**2022-02**	92	1
**2022-03**	1	8
**2022-03**	4	4
**2022-03**	11	1
**2022-03**	12	10
**2022-03**	22	6
**2022-03**	25	1
**2022-03**	28	1
**2022-03**	49	1
**2022-03**	76	1
**2022-03**	77	1
**2022-03**	87	2
**2022-03**	89	2
**2022-04**	1	23
**2022-04**	4	10
**2022-04**	12	27
**2022-04**	22	4
**2022-04**	75	1
**2022-04**	82	1
**2022-04**	87	2
**2022-04**	89	4
**2022-04**	102	2
**2022-05**	1	39
**2022-05**	2	1
**2022-05**	4	19
**2022-05**	12	44
**2022-05**	22	14
**2022-05**	28	2
**2022-05**	49	1
**2022-05**	58	1
**2022-05**	66	1
**2022-05**	77	2
**2022-05**	83	1
**2022-05**	87	5
**2022-05**	89	13
**2022-05**	103	1
**2022-05**	108	1
**2022-05**	122	1
**2022-05**	169	1
**2022-06**	1	31
**2022-06**	4	9

**2022-06**	6	1
**2022-06**	12	19
**2022-06**	22	12
**2022-06**	27	1
**2022-06**	28	4
**2022-06**	76	1
**2022-06**	81	1
**2022-06**	87	4
**2022-06**	89	6
**2022-07**	1	42
**2022-07**	4	21
**2022-07**	12	27
**2022-07**	18	1
**2022-07**	22	15
**2022-07**	25	1
**2022-07**	28	3
**2022-07**	48	1
**2022-07**	58	1
**2022-07**	68	1
**2022-07**	75	2
**2022-07**	81	3
**2022-07**	87	2
**2022-07**	89	8
**2022-07**	94	1
**2022-07**	95	1
**2022-07**	102	2
**2022-07**	104	1
**2022-08**	1	39
**2022-08**	4	20
**2022-08**	11	2
**2022-08**	12	24
**2022-08**	22	7
**2022-08**	28	1
**2022-08**	58	1
**2022-08**	73	1
**2022-08**	75	4
**2022-08**	81	2
**2022-08**	82	1
**2022-08**	87	6
**2022-08**	88	1
**2022-08**	89	3
**2022-08**	93	1
**2022-09**	1	19
**2022-09**	4	16
**2022-09**	11	2
**2022-09**	12	13

**2022-09**	22	8
**2022-09**	27	1
**2022-09**	28	1
**2022-09**	33	1
**2022-09**	44	1
**2022-09**	49	1
**2022-09**	58	1
**2022-09**	75	1
**2022-09**	77	1
**2022-09**	81	1
**2022-09**	82	2
**2022-09**	85	1
**2022-09**	87	3
**2022-09**	89	5
**2022-09**	104	1
**2022-10**	1	27
**2022-10**	4	20
**2022-10**	9	1
**2022-10**	12	9
**2022-10**	22	9
**2022-10**	28	2
**2022-10**	58	2
**2022-10**	73	1
**2022-10**	76	1
**2022-10**	82	1
**2022-10**	87	6
**2022-10**	89	5
**2022-10**	90	1
**2022-10**	94	1
**2022-10**	99	1
**2022-10**	102	1
**2022-10**	209	1
**2022-11**	1	43
**2022-11**	4	25
**2022-11**	11	2
**2022-11**	12	14
**2022-11**	22	8
**2022-11**	27	1
**2022-11**	28	1
**2022-11**	48	2
**2022-11**	49	2
**2022-11**	53	2
**2022-11**	58	1
**2022-11**	66	1
**2022-11**	75	4
**2022-11**	76	1

**2022-11**	77	3
**2022-11**	81	2
**2022-11**	82	4
**2022-11**	85	1
**2022-11**	87	4
**2022-11**	89	8
**2022-11**	102	1
**2022-11**	118	1
**2022-11**	183	2
**2022-12**	1	83
**2022-12**	4	11
**2022-12**	9	1
**2022-12**	11	1
**2022-12**	12	13
**2022-12**	22	13
**2022-12**	33	1
**2022-12**	44	2
**2022-12**	48	1
**2022-12**	58	1
**2022-12**	76	1
**2022-12**	82	1
**2022-12**	87	3
**2022-12**	89	1
**2022-12**	90	1
**2022-12**	95	1
